# An Empowerment-Based Physical Activity Intervention for Older People with Advanced Age-Related Macular Degeneration: An Exploratory Qualitative Case Study Design

**DOI:** 10.3390/jcm13133918

**Published:** 2024-07-04

**Authors:** Linn Håman, Jeanette Källstrand, Ing-Marie Carlsson, Andreas Ivarsson, Lars Kristén, Eva-Carin Lindgren

**Affiliations:** School of Health and Welfare, Halmstad University, SE-30118 Halmstad, Sweden; jeanette.kallstrand@hh.se (J.K.); ing-marie.carlsson@hh.se (I.-M.C.); andreas.ivarsson@hh.se (A.I.); lars.kristen@hh.se (L.K.); eva-carin.lindgren@hh.se (E.-C.L.)

**Keywords:** adapted physical activity, co-production, health, physical ability, social connectedness, visual impairment, well-being

## Abstract

**Background:** Age-related macular degeneration (AMD) is the most common cause of incurable visual impairment and impacts daily life. There are benefits of physical activity for people who are affected with AMD; however, living with AMD is associated with lower levels of physical activity and social isolation. The aim of this study was to explore how older people with AMD in Sweden experienced participation in a 6-month empowerment-based physical activity intervention and how it influenced their physical abilities. **Methods:** The participants were nine individuals with AMD aged 70–87 years. The intervention comprised physical and social activities in a group twice a week and individual health coaching on three occasions. The study was based on an exploratory qualitative case study design. **Results:** The findings showed two themes: created meaningfulness in life and creative and playful ways to develop body movements. The findings also showed improved muscle strength after the intervention. **Conclusions**: The findings showed that participants had increased social connectedness, improved physical self-efficacy and physical ability, as well as improved muscle strength. The empowerment process of the intervention was appreciated by the participants and challenged them to participate in physical activity offered by the municipality for older individuals.

## 1. Introduction

Over time, the global population has been aging [1], and increasing age entails several risk factors, including impaired vision [2]. Research has shown that the leading cause of visual impairment in older people is age-related macular degeneration (AMD), which impacts central vision and is recognized as the third most prevalent cause of vision impairment and legal blindness among those aged 60 years and above [3,4]. AMD occurs in two forms, wet (neovascular) and dry (atrophic), which could both impact one or both eyes without causing pain [4,5]. Advanced AMD affects the performance of daily activities and causes emotional impacts, such as frustration and fear of blindness [6].

AMD has also made older individuals less likely to participate in social activities and made them more isolated [7]. This group made fewer excursions from home than individuals with normal vision [8], which was crucial for their health and well-being [9]. Approaches that enhance social connection among older adults have been essential [10], including group-based physical activity interventions [11].

Physical activity might have decreased the risk of AMD development and progression [12] and shows a protective association with both early and late AMD [13]. Despite these advantages, research has shown that several eye conditions, including AMD, were associated with lower levels of physical activity [12,14]. Individuals with worse vision tended to engage in lower levels of physical activity [8,12,15], and individuals with late AMD spent less time in moderate-to-vigorous physical activity (MVPA) than those with early AMD or without AMD [12,16]. Previous research has shown that various barriers hinder individuals with sight loss from being physically active or increasing their level of physical activity [17]. For instance, fear of falling [18], lack of social support, safety concerns, and low confidence contribute to the difficulty in being active [17].

Furthermore, these barriers are interconnected with health factors such as physical fitness levels, health conditions, and mental and emotional well-being [17]. Additionally, unhealthy daily lifestyle movement patterns were also more common among individuals with AMD, which underscores the significance of promoting physical activity within this group [16]. Older individuals with sight loss have been shown to be diverse and, therefore, require different opportunities for physical activity based on their preferences, previous experiences, and levels of participation [17].

A systematic review showed that resistance training was beneficial for improving muscle strength and physical performance in older people [19]. An intervention study showed that sedentary older adults who participated in a posture–balance motricity and health education program exhibited notably greater progress in basic motor abilities [20]. Interventions that included group exercise, individualized consultation, and social support during physical activity have been associated with improvements in both physical activity and loneliness [21]. Additionally, research indicated that participation in sports promotes social interaction, community, and support among older adults, reducing feelings of loneliness and social isolation and contributing to increased well-being [22]. Thus, scholars have considered incorporating social activities into physical activity interventions [21] for individuals with AMD.

Performing a physical activity with someone else has been shown to be associated with more pleasure than performing the activity alone [23]. Additionally, given that not all individuals with AMD have had a physically active lifestyle, it could be important to listen to their voices regarding their needs and wishes by adopting a bottom-up approach in an intervention. A bottom-up approach can enable individuals to gain power in the decision-making process, influencing the content of the intervention, which is in accordance with empowerment. Empowerment focuses on relationships with others and the transfer of power with outcomes, such as emancipation [24], engaging in discussions, decision-making, and acting for oneself. An empowerment-based approach can be crucial in individuals’ everyday lives and support them in taking control of the factors that influence their health [25].

Participating in a physical activity intervention with others in a similar situation, where people can exchange experiences, be social and support each other, can promote social connectedness, reduce loneliness [11], and create empowerment [24]. Nevertheless, scholars have argued that low levels of participation in physical activity in older adults suggests that their wants and needs are not being met in the promotion of physical activity [26]. This study provides knowledge of an empowerment-based physical activity intervention (EPI) for older individuals with AMD. Listening to the voices of individuals with AMD is crucial, since their perceptions and experiences could facilitate the development and implementation of physical activity interventions. The aim of this study was to explore how older people with AMD in Sweden experienced participation in a 6-month EPI and how the intervention influenced their physical abilities..

## 2. Material and Methods

### 2.1. Design

This study was produced through an exploratory qualitative case study design [27] aimed at exploring how older people with advanced age-related macular degeneration experienced their participation in an Empowerment-Based Physical Activity Intervention and how the intervention influenced their physical ability. An exploratory case study design can be used when exploring a phenomenon in context, using one or more data collection methods, and decrypting a case in-depth [27]. such as the EPI. This study used qualitative data sources supported with quantitative data.

### 2.2. The Empowerment-Based Physical Activity Intervention

The EPI was part of a larger Interreg project funded by Öresund–Kattegat–Skagerrak European Regional Development (NYPS-20293225) with cross-border collaboration between Sweden and Denmark named *Can you see the future?* This study focused on an EPI that was carried out in a community of about 100,000 inhabitants in the southwest of Sweden [28]. A reference group was established and consisted of visually impaired people and professionals such as low-vision therapists, ophthalmic nurses, Halmstad Municipality activity centres, Halland’s Sports Movement, and Parasport Association employees. The reference group was invited to events where the focus was on describing the project idea, obtaining feedback on the implementation and evaluation of the intervention, and identifying potential pitfalls, challenges, and ways to overcome them [28].

The theoretical framework of this research project was based on salutogenic approaches [29] focusing on health, and not on disease (i.e., AMD). The EPI was also based on empowerment as a health-promoting strategy in which both top-down and bottom-up approaches were combined [30]. A top-down approach was applied when the researchers initiated the intervention, and a bottom-up approach was employed to enable the participants to gain power in the decision-making process. Thus, the EPI partly had a co-constructed design, where the participants took an active part in the planning and implementation of the EPI, which ensured that the intervention was tailored to the participants’ needs and wishes. This supported the participants in taking control of the factors that influenced their health [25].

The goal of the EPI was to promote health and physical activity and to increase the participants’ well-being, vision-related quality of life, and social connection [28]. To accomplish this, activity coaches interacted with the participants twice a week and one hour after workouts. This was also accomplished through three individual health coaching sessions (30 min), which were conducted with each participant at the beginning, middle, and end of the intervention.

The health coaching was carried out by two of the activity coaches and took place individually with all participants. It was a structured conversation based on the T-GROW (topic, goal, reality, option, and will) model [31]. The health coaching sessions were structured around open-ended questions exploring various options, reframing, expressions of empathy, summaries, and goal setting [32,33]. The conversation focused on the participants’ goals for participating in the EPI, how to achieve these goals, current situations, obstacles that may arise, and how the EPI could be a support. The formulation of their needs, potential solutions, and necessary actions to solve this was in line with an empowerment process [25].

### 2.3. Activities

The activities in the EPI consisted of physical activities based on the participants’ abilities, social gatherings, and health coaching. Coffee, sandwiches, and fruit were provided at the physical activities and social gatherings, which were conducted two days per week for six months. The four activity coaches were two researchers (LH and LK) and two students who led the physical activities, which lasted for one hour per session.

The physical activities were conducted at Halmstad University’s health lab and gymnastics hall. These activities consisted of different forms of training aimed at promoting the participants’ balance, strength, and fitness, such as balance training, strength training, mat curling, and various adapted ball games for people with disabilities. There was also collaboration with local sports associations, activity leaders in the municipality, and the National Association of the Visually Impaired. This collaboration involved leaders from various sports associations holding a few activity sessions for the participants and informing them about, and inviting them to, their respective organizations for activities such as table tennis, mat curling, various adapted ball games, sitting yoga, and sitting exercises.

The variety of activities contributed to participants having the opportunity to test both familiar and new forms of physical activity. One important aspect when planning the activities was that the participants would always be able to practice the activities based on their own functional ability instead of considering their various disabilities as obstacles. The physical activity sessions were followed by an hour of socializing with coffee provided. These sessions consisted of a variety of informal conversations where participants could socialize and share experiences, and some of the sessions consisted of organized conversations such as information on AMD followed by question-and-answer sessions.

### 2.4. Participants

Individuals who were ≥65 years old and had advanced dry AMD were invited to participate in the EPI. This meant that participants were most likely to have had the experience needed to offer valuable insights and information-rich data [34]. The inclusion criteria included a diagnosis of advanced dry AMD in one or both eyes and a visual acuity of no more than 0.3 in the best eye (normal visual acuity is 0.8–1.0). Other inclusion criteria were contact with a low-vision clinic, living at home, ability to walk independently, and ability to freely communicate in Swedish. Candidates were excluded if engaging in high-intensity physical activity more than two days per week or if experiencing severe health risks during physical activity.

Recruitment was conducted in collaboration with a low-vision clinic in Southwestern Sweden. Staff in the low-vision clinic in Halland County were informed about the study’s purpose and structure. The staff identified potential candidates according to patient records, and 66 individuals with advanced dry AMD were identified, of whom 46 met the inclusion criteria. The staff informed the identified candidates either when visiting the clinic or by phone. One of the participants was recruited via one of the researchers after hearing about the study and was asked to participate. Candidates who gave permission were contacted and given more information by a researcher (JK). In case of continued interest, the candidate gave written consent and agreed to participate in the EPI.

In total, 11 individuals with AMD were recruited to participate in the EPI (9 women, and 2 men). Two of the participants dropped out after a month, while another took a break for about two months but then came back. The reasons for dropping out and taking a break were personal. The final sample consisted of 7 women and 2 men aged 70–87 years with advanced AMD (Table 1).

### 2.5. Data Collection

Data were collected in four ways: demographic data, a sit-to-stand test (30STS) test protocol, participant observations during the EPI, and focus group interviews (FGIs). The main data collection consisted of the participant observations and FGIs.

#### 2.5.1. Demographic Data

Demographic data were collected before the EPI started. One of the researchers (JK) collected the participants’ demographic data (age, sex, marital status, and housing).

#### 2.5.2. Sit-to-Stand Test (30STS)

The Sit-to-Stand Test was conducted before the intervention started, after four months, and at the end of the EPI (six months). Two of the researchers (LH and LK) conducted most of the 30STS tests with the support of other members of the research team. The 30STS [35] measured lower-extremity muscle strength. The participants were encouraged to complete as many full stands as possible within 30 s with the arms crossed in front of the chest and the feet parallel. The score was the total number of stands completed within that time. A practice test was performed before timekeeping started. A chair with a standardized height (45 cm) and a stopwatch were used. The test had good validity and reliability in measuring bone strength in older individuals [35].

#### 2.5.3. Observation

The observations allowed us to understand central factors in the EPI and the meaning that participants ascribed to the EPI [36]. Participant observations were performed at each intervention session (two days a week for six months). The observations were conducted by the four activity coaches, from the same culture as that of the participants. Field notes of the observations were written continuously in connection with the intervention activities. The field notes covered what the participants talked about during the EPI and their interactions with each other related to the experiences with it.

The notes were both descriptive in nature and included quotes expressed by participants. The participant observations enabled informal conversations, the development of in-depth knowledge regarding experiences within the group and meaning-making regarding physical and social activities [37]. The field notes served as a complement to the FGIs to enable better understanding of the experiences.

#### 2.5.4. Focus Groups Interview

FGIs were conducted at the end of the EPI. The FGIs were chosen since they encouraged discussion and were a suitable method when studying attitudes and values [38]. One week after the end of the EPI, FGIs were conducted to gain insight into how older people with AMD experienced participation in the EPI. All nine participants took part in the FGIs (four participants in one group and five in another). Four of the researchers carried out the FGIs. Two of them acted as moderators (IMC and ECL), and two of them acted as observers (JK and LK).

The FGIs included questions regarding what it meant for participants to partake in the EPI and how they could maintain their physical activity after the EPI. Four researchers (two in each focus group) led the discussions and asked follow-up questions to allow for detailed discussion. One of the researchers acted as a moderator, and the other was an observer. The FGIs were conducted in a conference room at Halmstad University, lasted approximately 50–60 min, and were audio-recorded and transcribed verbatim.

### 2.6. Qualitative Analyses

A thematic analysis [39,40] was employed to thematize the qualitative data sources. First, two of the researchers (LH and ECL) read all transcripts several times to gain an overall view of the experiences. Succinct codes were generated on important features of the data that addressed the aim, and the codes were examined to develop broader patterns (tentative themes). LH and ECL reviewed and discussed the tentative subthemes in relation to the coded data. In this phase, the tentative themes were further developed and arranged into two themes.

A detailed analysis of each theme was performed by determining the storyline and choosing informative names of the two themes. LH and ECL weaved together the analytic narrative and contextualized the analysis by moving between the themes and literature to interpret the experience regarding the EPI. All researchers reviewed the interpretation of the empirical findings. Although these analytical phases were described sequentially, they actually involved a recursive process and a moving back and forth between the different phases.

### 2.7. Statistical Analyses

One of the researchers (AI) conducted the quantitative data analysis. Quantitative data were analysed using JASP (version 0.18.1.0). Descriptive statistics were expressed as means ± standard deviations (SD) for continuous data. The differences between the start and end of EPI were analysed using Bayesian paired-sample *t*-tests with default priors and compared to the null model. Bayes factors (BFs) between 1 and 3 were considered to indicate weak evidence for the alternative hypothesis, BFs between 3 and 10 were considered as moderate evidence for the alternative hypothesis, and BFs greater than 10 were considered as strong evidence for the alternative hypothesis [41].

### 2.8. Ethics

The participants received oral and written information about the study and were informed that participation was voluntary and that they could interrupt without explanation or without affecting their continued care. In addition, the participants signed a written informed consent form to participate. The participants also received information regarding personal data management according to the General Data Protection Regulation (GDPR). Confidentiality was ensured, and there was assurance that only the research group had access to data. The study was conducted according to the guidelines of the Declaration of Helsinki [42] and approved by the Swedish Ethical Review Authority (EPN 2021/02877, 12 December 2020).

## 3. Results

The aim of this study was to explore how older people with AMD in Sweden experienced participation in a 6-month EPI and how the intervention influenced their physical abilities. First, the qualitative findings are presented. These findings showed two overarching themes: created meaningfulness in life and creative and playful ways to develop body movements.

### 3.1. Created Meaningfulness in Life

During the COVID-19 pandemic, the participants’ everyday lives created distance from children, friends, and social life. Much time was spent at home without direct social contact. For some, loneliness was more evident as they were living alone without a partner. The EPI has been described as contributing to meaningfulness, and all participants looked forward to meeting each other at the gatherings and longed for the activities. Due to the long period of social distance, the participants expressed that having a goal (participating in the EPI) for the day was nice, while one said that “the program is the best thing that happened in my life” (field note).

Meaningfulness was linked to the activities in the EPI and contributed to a new dimension in life. One said, ”I no longer talk about diseases with my family and relatives. Instead, we talk about what I do here” (field note). Created meaningfulness in life consisted of the following subthemes: (1) being part of a supportive and caring group and (2) being included, inspired, and having fun.

#### 3.1.1. Being Part of a Supportive and Caring Group

Each activity started with a mixed session where everyone had to introduce themselves by name. Since the participants had a visual impairment, the presentation made it easier to recognize who was there. The participants expressed that the EPI contributed to a feeling of being part of a group that was meaningful, since it was possible to socialize and make new friends. At one point, this was expressed as follows: “I’m so glad my son got me here today; we’re like family!”

The participants expressed that they looked forward to the social gatherings, among other things, and described the EPI as one of the highlights of the week. After a while, the participants felt close to the group, partially because of the coffee breaks after the activities. The participants reported that they felt cared for by the other participants and also cared for the others in the group. For instance, they exchanged experiences with each other about living with AMD, giving each other advice on how everyday life can be made easier with technical functions that enable people with visual impairments to send messages and receive news. If someone was experiencing something challenging at home, others could offer advice about thinking differently.

Gradually, the participants also helped each other to carpool to the EPI so everyone could attend. Another indication of the feeling of being a part of a group was when the participants felt comfortable joking and inviting themselves. Some participants and their partners also started socializing outside the EPI with other participants in the EPI. One said, “We have visited each other’s homes, and our partners hang out and shop together” (FGI 1).

#### 3.1.2. Being Included, Inspired, and Having Fun

The participants expressed enjoyment of the EPI due to a sense of being included. One participant said, “Everyone can participate, even if we are a little different.” In FGI 1, participants “appreciated that the activity coaches waited for us at the door and always greeted us with a smile.” The other participants also expressed that they felt understood after meeting others with the same diagnosis. One said, “It’s not so much fun to be visually impaired, but it was fun to meet others in the same situation and that the activities were tailored for us” (FGI 2). The participants also appreciated when the activity coaches understood their visual impairment.

They also felt seen and prioritized by the activity coaches. One of the reasons was that the activities were individually tailored to each participant’s functional ability. For example, in one physical activity session, a participant expressed, “It’s so much fun that it’s possible to arrange so that everyone can participate, so you don’t have to stand by and watch while others are active.” Another participant replied, “Yes, you often must do that otherwise. Everything can be solved here. We have learned that now.”

On several occasions, the participants also reported being inspired and satisfied with the activities in the intervention. The participants experienced joyful body movements that involved much more imaginative physical activity than they had engaged in before, which was inspiring. It was also inspiring to test new activities to cooperate in teams, and they wanted to be able to repeat completed activities on more occasions. On several occasions, several of the participants were observed to perform physical exercises that used to be carried out during the activities in the EPI even before the activity started.

There was always a good atmosphere in the group during the activities, and participants encouraged and motivated each other. During one of the physical activity sessions, one participant said to another, “You inspired me when you started dancing when we did the slalom between the cones, so I couldn’t resist. The music inspired me to dance.” One of the participants explicitly described that his physical development during the program contributed to motivation for continued exercise, and several participants discussed a desire to continue to perform physical activity together after the EPI ended.

In addition, participants showed joy and gained extra motivation when receiving praise from activity coaches and other participants. Several participants also stated that they received inspiration from each other and the activity coaches to perform physical activity in different contexts after the EPI. One participant expressed gratitude that a person from the municipality was invited to the EPI to inform them about possible activities offered by the municipality that they could join after the end of the EPI. In both informal conversations and FGIs, the participants expressed that it had been a big challenge to attend such activities themselves before participation in the EPI. One of them said, “When they sent out information sheets before, I thought I couldn’t participate. I couldn’t handle it, but it was good that she (a woman from the municipality) could explain more.”

### 3.2. Creative and Playful Ways to Develop Body Movements

During the EPI, the participants tried various tailored physical activities that made them discover their ability to develop their movement capabilities and skills. The activities were designed based on the participant’s needs and wishes, as well as to allow them to experience creative activities to develop their movement capabilities and skills in order to better manage everyday life. The playfulness of the activities contributed to the participants’ abilities to perform more challenging movements than expected. Several times, the participants expressed satisfaction and happiness with their development and the activities in the EPI by describing and demonstrating their movement capabilities and skills. Creative and playful ways to develop body movements showed two subthemes: (1) improving physical ability, strength, and balance and (2) daring to push boundaries.

#### 3.2.1. Improving Physical Ability, Strength, and Balance

Participants in the study expressed improved physical ability, strength, and balance due to participation in the EPI. The participants expressed satisfaction with their enhancement, which was frequently communicated by describing and showing their improvement in movement capabilities and skills during the program. After a month, one of the participants proudly demonstrated her achievement in successfully standing up from a chair without needing any external assistance or aids. Another participant reported, “I practiced walking (independently) in the gymnastics hall. The activity walked beside me, then I walked independently, without my walker. (…) I also exercise a little at home” (FGI 2). This improvement was evident during the physical activity sessions since the participant made clear developments during the EPI.

In the first session, one participant partook in the activities by sitting in a wheelchair. After a few weeks, the participant progressed to rising from the wheelchair to a standing position. After that, the participant replaced the wheelchair with a rollator/walker. At the end of the EPI, the participant could walk independently next to one of the activity coaches. During the independent walk, the participant described fantasizing about being on a catwalk in an adorable dress and that this sense of independence had returned her “spark of life.”

The participants appreciated the elements of balance exercises in each session. Classic balance exercises, such as standing without holding aids and standing on one leg, were interspersed with more playful activities. For instance, one activity that included playful balance training for both legs was playing walking football. Another playful activity was carrying a bean bag on their head and walking without dropping the bag. The participants appreciated this variation in activities. Several times, the participants described that it was pleasant to move and work with the whole body; they stated that they had become satisfied with themselves and felt well-being after the activities.

During an activity where the participants played soccer, another participant expressed, “You learn by trying—for example, I have never thought before that you can stop the ball before you shoot it.” The participants also described a notable reduction in physical complaints. For instance, one of the participants said that she no longer needed treatment from a physiotherapist due to her participation in the EPI. Another participant described that this clear improvement increased their willingness to train with a clear goal, such as walking in the home without a rollator/walker.

Moreover, the participants also emphasized the importance of improving their physical ability to achieve continuous independence. For instance, one of them said, “In the health coaching session, she (the activity coach) asked what my goal is, and my goal is to be able to take care of myself as long as I can live by myself. It is my most important goal to be able to manage myself.” Another participant replied, “Yes, it is at the top of the list” (FGI 2).

#### 3.2.2. Daring to Push Boundaries

The feeling of security was crucial for the participants in pushing their boundaries. The participants also described that the EPI, characterized by physical activities tailored to individual physical conditions and facilitated by multiple activity coaches, engendered a profound sense of security. During one of the sessions, one of the participants said, “That’s precisely what is so good about this, that you dare to do things you wouldn’t have done otherwise, even if you’re old and have your obstacles.” Several participants reported surprise that they could do more movements and activities than expected. One participant expressed happiness during a physical activity session when pushing boundaries: “It’s so much fun to try something you haven’t done before; you constantly surpass yourself.” Another participant expressed that it was challenging to keep balance during the exercises but thought it was a good exercise: “Not everything in life is easy, but why take the easy way out? This was a good challenge!”

Participants challenged their physical ability by employing fitness equipment, such as free weights and resistance bands. This approach enabled participants to discern what they could do and their limitations, as exemplified by the following statement: “It’s fun to see your limitations physically but also discover everything you can do.” Since the participants could observe peers pushing their boundaries and receiving encouragement from each other and the activity coaches, they voiced an increased willingness to explore and test their physical ability themselves or by competing playfully. One participant noted a discernible enhancement in physical ability during the EPI and stated at one point: “I feel successful when I repeat the exercises.” One of the male participants said with a twinkle in his eye: “We will become real athletes.”

### 3.3. Statistical Results

A total of nine of the participants had data from the start and end of EPI and were included in the statistical analyses. For the sit-to-stand test, the Bayesian paired-sample *t*-test showed strong evidence for the alternative hypothesis indicating that the participants showed higher values at the end of EPI (M = 12.11, SD = 4.43) in comparison to the start of the intervention (M = 8.17, SD = 4.43; t = 3.89, BF = 11.75).

## 4. Discussion

This study shows how older people with AMD experience participation in an EPI and how the intervention influences their physical activity andability. The results reveal two overarching themes: created meaningfulness in life, and creative and playful ways to develop body movements. One of the main findings indicates that the EPI design promotes social connectedness. since participants describe close bonds with the other individuals with AMD, i.e., being part of a supportive and caring group. In addition, the participants report that the EPI decreased their loneliness, since they met new friends and did not need to be socially isolated.

Research shows that older individuals living with AMD experience more isolation than others [7]. In a scoping review, social connectedness is described as the opposite of loneliness [43]. It is possible that the feeling of social connectedness was reinforced after the COVID-19 pandemic. Even if Sweden had a more liberal strategy regarding social distancing during the COVID-19 pandemic in comparison to other countries, it was evident that many community-dwelling older people aged 70 and above and those with underlying illnesses in Sweden were recommended to self-isolate [44]. Internationally, scholars argue that loneliness and social isolation frequently occur among older people [45], including those in Sweden [44].

Research shows that subjective evaluation of having meaningful, close, and constructive relationships with others is central to social connectedness [43]. This is also evident in group-based physical activity interventions for older people [11]. The EPI contributed to creating opportunities for older people to leave their homes and socialize with others, which is particularly important since people with AMD make fewer excursions from home compared to individuals with normal vision [8]. Time out of home is essential for older people’s health and well-being, e.g., [9] and is also related to social connectedness [46]. Being part of a supportive and caring group also include feeling cared for by the other participants and caring for the others in the group. For instance, participants share experiences and offer each other advice in their daily lives. Similar results are found in a study that included motivational group meetings [11].

Another finding indicates that the design of the EPI creates a sense of being included, since all activities were tailored to the participants’ wishes and needs so that everyone could participate according to their own movement capabilities and skills. It was possible to tailor the physical activity and encourage participants to develop individually, as the activities were led by four activity coaches for a small group of nine participants. The sit-to-stand task test also helped assess balance and fall risk among participants, allowing activity coaches to tailor activities for the participants. A systematic review shows the importance of tailoring interventions to participants. Matching the individual interests of older adults seems particularly important [47], and so does tailoring interventions when promoting pleasurable activities [23].

Moreover, staff from the municipality and sports organizations were invited to test the participants’ activities in order to encourage them to remain physically active in other organized physical activities after the EPI. Several participants also state that they received inspiration from each other and the activity coaches to perform physical activity in different contexts after the EPI, which they appreciated and started to implement. Interventions starting with tailored guidance that then provide ongoing support have been shown to improve participation in physical activity among older adults [47], of which the EPI can be seen as an example.

This research is designed to listen to the participants’ voices when designing strategies to promote physical activity. As observed, participants express a sense of inspiration and having fun when participating in the EPI. Promoting fun rather than just health and fostering social interaction within interventions can lead to older adults’ enjoyment of physical activity [26]. Likewise, engaging in physical activity with others is linked to greater pleasure compared to engaging in the activity alone [23]. In this study, participants express joy and gain extra motivation from other participants when receiving praise from the activity coaches.

One of the most important goals of participating in the EPI for the participants is that they could take care of themselves and live by themselves, thus becoming independent from others. Therefore, they needed to attain better motor competence. Perceived motor competence is generally defined as an individual’s assessment of their movement capabilities and skills to perform a specific skill or movement [48]. The findings show that the participants in the EPI perceived improved physical ability, strength, and balance with improved movement capabilities and skills.

The results from the sit-to-stand test show improvement in functional ability during the EPI. This improvement supports the findings from the qualitative data, where the participants expressed experienced improved physical abilities. This can be seen as increased (physical) self-efficacy [49]. According to Tengland [25], self-efficacy is a specific or narrow goal within empowerment, and this mental resource is believed to represent either actual increased control or a causal contributor to increased control.

The participants experienced a reduction in physical complaints, and one of them did not need treatment from a physiotherapist anymore. Maintaining functional ability is paramount for healthy aging as it enables individuals to maintain health and well-being [1]. Scholars show that, if older adults perceive their movement capability as higher, they have more confidence in movement situations and a greater likelihood of continued engagement in physical activity [48].

Findings also show that the participants were proud and satisfied after each session. They also appreciated the balance exercises and the activity coaches doing playful exercises. Similar findings are evident in a review [26], which found that older adults are often motivated to engage in physical activity when the activity promotes fun rather than just health and that intrinsic enjoyment was apparent in a wide range of physical activity. Furthermore, increased feelings of well-being were experienced immediately after engaging in physical activity. Findings in this study show that the participants were surprised that they could do more than expected, which is also found in the same review [26].

Our findings also demonstrate that the participants dared to push their boundaries because they felt secure that the physical activities were adapted to their physical conditions and because several activity coaches could care for them. Having four activity coaches for a small group of nine participants might have increased the participants’ level of (physical) self-efficacy [49] daring them to challenge themselves and thus improve physical abilities. It is certainly resource-intensive to have several activity coaches, but this empowerment-based strategy may be particularly important if the intention is to encourage participants to improve movement capabilities and skills, so that they can become more independent and improve their well-being. This is important for motivating participants to participate in community-based physical activities after the intervention.

### 4.1. Conclusions

This study provides new insights into how both older individuals experience an EPI and how the design of the intervention influenced the participant’s physical ability and agency to act. The qualitative findings show that participants experience increased social connectedness, improved physical self-efficacy, and physical ability. The quantitative results showed improved muscle strength between the start and the end of the EPI. The participants highly appreciate the empowerment processes of the intervention design, such as the involvement in health coaching and the decision-making process, adapted and tailored activities, social events, and the encouragement and support from the activity coaches. This also motivates them to participate in physical activity for older individuals offered by the municipality.

### 4.2. Limitations and Strengths

One of the methodological limitations was that our findings are based on a small sample of older Swedish individuals with AMD and with gender imbalance, which does not reflect the diversity of the AMD population in Sweden. Therefore, the findings can only be transferred to similar groups of older individuals with AMD. However, it was not our intention to generalize the findings from this study. Future research could benefit from conducting a randomized controlled trial (RCT) and then including relevant physical health metrics, such as cardiovascular health or overall physical fitness. A strength of the study was that there was cross-checking with the participants regarding the interpretation of their experiences to ensure that the true meaning was reflected.

## Figures and Tables

**Table 1:** Overview of participants.

Participant	Age	Sex	Marital Status	Housing
1	85	Male	Married	House
2	81	Female	Married	House
3	70	Female	Married	Condominium apartment
4	87	Female	Widow	Rental apartment
5	86	Male	Married	House
6	80	Female	Widow	Condominium apartment
7	82	Female	Married	Condominium apartment
8	81	Female	Widow	Condominium apartment
9	79	Female	Divorced	Rental apartment

## Data Availability

All data will be coded so that only relevant researchers can access it. Data will be locked in a safe at the university, away from the code key, which will be locked in another safe. Research material is saved for ten years after the research principal completes the project. Only researchers who belong to the project will have access to data. All information that can reveal the identity of the researchers will be pseudonymized.

## References

[1] United Nations (2023). World Social Report 2023: Leaving No One behind in an Ageing World.

[2] Maier A., Riedel-Heller S., Pabst A., Luppa M. (2021). Risk factors and protective factors of depression in older people 65+. A systematic review. PLoS ONE.

[3] Mitchell P., Liew G., Gopinath B., Wong T.Y. (2018). Age-related macular degeneration. Lancet.

[4] Taylor D.J., Smith N.D., Binns A.M., Crabb D.P. (2018). The effect of non-neovascular age-related macular degeneration on face recognition performance. Graefes Arch. Clin. Exp. Ophthalmol..

[5] Bhutto I., Lutty G. (2012). Understanding age-related macular degeneration (AMD): Relationships between the photoreceptor/retinal pigment epithelium/Bruch’s membrane/choriocapillaris complex. Mol. Asp. Med..

[6] Sivaprasad S., Tschosik E.A., Guymer R.H., Kapre A., Suñer I.J., Joussen A.M., Lanzetta P., Ferrara D. (2019). Living with Geographic Atrophy: An Ethnographic Study. Ophthalmol. Ther..

[7] Dillon L., Gandhi S., Tand D., Liew G., Hackett M., Craig A., Mitchel P., Keay L., Gopinath B. (2021). Perspectives of people with late age-related macular degeneration on mental health and mental wellbeing programmes: A qualitative study. Ophthalmic Physiol. Opt..

[8] Sengupta S., Nguyen A.M., van Landingham S.W., Solomon S.D., Do D.V., Ferrucci L., Friedman D.S., Ramulu P.Y. (2015). Evaluation of real-world mobility in age-related macular degeneration. BMC Ophthalmol..

[9] Petersen J., Austin D., Mattek N., Kaye J. (2015). Time Out-of-Home and Cognitive, Physical, and Emotional Wellbeing of Older Adults: A Longitudinal Mixed Effects Model. PLoS ONE.

[10] Suragarn U., Hain D., Pfaff G. (2021). Approaches to enhance social connection in older adults: An integrative review of literature. Aging Health Res..

[11] Franke T., Sims-Gould J., Nettlefold L., Ottoni C., McKay H.A. (2021). “It makes me feel not so alone”: Features of the Choose to Move physical activity intervention that reduce loneliness in older adults. BMC Public Health.

[12] Ong S.R., Crowston J.G., Loprinzi P.D., Ramulu P.Y. (2018). Physical activity, visual impairment, and eye disease. Eye.

[13] McGuinness M.B., Le J., Mitchell P., Gopinath B., Cerin E., Saksens N.T.M., Schick T., Hoyng C.B., Guymer R.H., Finger R.P. (2017). Physical Activity and Age-related Macular Degeneration: A Systematic Literature Review and Meta-analysis. Am. J. Ophthalmol..

[14] Heinemann M., Welker S.G., Li J.Q., Wintergerst M.W.M., Turski G.N., Turski C.A., Terheyden J.H., Mauschitz M.M., Holz F.G., Finger R.P. (2019). Impact of visual impairment on physical activity in early and late age-related macular degeneration. PLoS ONE.

[15] Lindsay R.K., Di Gennaro F., Allen P.M., Tully M.A., Marotta C., Pizzol D., Gorely T., Barnett Y., Smith L. (2021). Correlates of Physical Activity among Adults with Sight Loss in High-Income-Countries: A Systematic Review. Int. J. Environ. Res. Public Health.

[16] Loprinzi P., Swenor B., Ramulu P. (2015). Age-Related Macular Degeneration Is Associated with Less Physical Activity among US Adults: Cross-Sectional Study. PLoS ONE.

[17] Phoenix C., Griffin M., Smith B., Howe D.P. (2015). Physical activity among older people with sight loss: A qualitative research study to inform policy and practice. Public Health.

[18] Nguyen A., Arora K., Swenor B., Friedman D., Ramulu P. (2015). Physical activity restriction in age-related eye disease: A cross-sectional study exploring fear of falling as a potential mediator. BMC Geriatr..

[19] Lai C.C., Tu Y.K., Wang T.G., Huang Y.T., Chien K.L. (2018). Effects of resistance training, endurance training and whole-body vibration on lean body mass, muscle strength and physical performance in older people: A systematic review and network meta-analysis. Age Ageing.

[20] Bernard P., Blain H., Gerazime A., Maurelli O., Bousquet J., Ninot G. (2018). Relationship between a three-month physical conditioning “posture-balance-motricity and health education” (PBM-HE) program on postural and balance capacities of sedentary older adults: Influence of initial motor profile. Eur. Rev. Aging Phys. Act..

[21] Ahn J., Falk E.B., Kang Y. (2024). Relationships between physical activity and loneliness: A systematic review of intervention studies. Curr. Res. Behav. Sci..

[22] Gayman A.M., Fraser-Thomas J., Dionigi R.A., Horton S., Baker J. (2017). Is sport good for older adults? A systematic review of psychosocial outcomes of older adults’ sport participation. Int. Rev. Sport. Exerc..

[23] Cabrita M., Lousberg R., Tabak M., Hermens H.J., Vollenbroek-Hutten M.M.R. (2017). An exploratory study on the impact of daily activities on the pleasure and physical activity of older adults. Eur. Rev. Aging Phys. Act..

[24] Leyshon S. (2002). Empowering practitioners: An unrealistic expectation of nurse education?. J. Adv. Nurs..

[25] Tengland P.A. (2012). Behavior change or empowerment: On the ethics of health-promotion strategies. Public Health Ethics.

[26] Devereux-Fitzgerald A., Powell R., Dewhurst A., French D.P. (2016). The acceptability of physical activity interventions to older adults: A systematic review and meta-synthesis. Soc. Sci. Med..

[27] Yin R.K. (2018). Case Study Research: Design and Methods.

[28] Lindgren E.C., Källstrand J., Johansson P., Kristén L., Håman L., Ivarsson A., Carlsson I.M. (2023). Empowerment-Based Physical Activity Intervention for People with Advanced Dry Age-Related Macular Degeneration: Mixed-Methods Protocol. Int. J. Environ. Res. Public Health.

[29] Antonovsky A. (1996). The salutogenic model as a theory to guide health promotion. Health Promot. Int..

[30] Braunack Mayer A., Louise J. (2008). The ethics of community empowerment: Tensions in health promotion theory and practice. Promot. Educ..

[31] Downey M. (2014). Effective Modern Coaching: The Principles and Art of Successful Business Coaching.

[32] Heimendinger J., Uyeki T., Andhara A., Marshall J.A., Scarbro S., Belansky E., Crane L. (2007). Coaching Process Outcomes of a Family Visit Nutrition and Physical Activity Intervention. Health Educ. Behav..

[33] Olsen J. (2014). Health Coaching: A Concept Analysis. Nurs. Forum.

[34] Denscombe M. (2010). The Good Research Guide For Small-Scale Social Research Projects.

[35] Jones C.J., Rikli R.E., Beam W.C. (1999). A 30-s Chair-Stand Test as a Measure of Lower Body Strength in Community-Residing Older Adults. Res. Q. Exerc. Sport.

[36] Atkinson P., Coffey A., Delamont S., Lofland J., Lofland L. (2007). Handbook of Ethnography.

[37] Swain J., King B. (2022). Using Informal Conversations in Qualitative Research. Int. J. Qual. Methods.

[38] Bryman A. (2016). Social Research Methods.

[39] Braun V., Clarke V. (2019). Reflecting on reflexive thematic analysis. Qual. Res. Sport. Exerc. Health.

[40] Braun V., Clarke V. Doing Reflexive TA. University of Auckland, New Zealand, NS. https://www.thematicanalysis.net/doing-reflexive-ta/.

[41] van Doorn J., van den Bergh D., Böhm U., Dablander F., Derks K., Draws T., Etz A., Evans N.J., Gronau Q.F., Haaf J.M. (2021). The JASP guidelines for conducting and reporting a Bayesian analysis. Psychon. Bull. Rev..

[42] World Medical Association (2013). World Medical Association Declaration of Helsinki: Ethical Principles for Medical Research Involving Human Subjects. JAMA.

[43] O’Rourke H., Sidani S. (2017). Defintions, determinants, and outcomes of social connectedness for older adults: A scoping review. J. Gerontol. Nurs..

[44] Aartsen M., Rothe F. (2023). The Impact of the COVID-19 Pandemic on Social Isolation and Loneliness: A Nordic Research Review.

[45] Hwang T., Rabheru K., Peisah C., Reichman W., Ikeda M. (2020). Loneliness and social isolation during the COVID-19 pandemic. Int. Psychogeriatr..

[46] Morgan T., Wiles J., Park H.J., Moeke-Maxwell T., Dewes O., Black S., Williams L., Gott M. (2021). Social connectedness: What matters to older people?. Ageing Soc..

[47] Zubala A., MacGillivray S., Frost H., Kroll T., Skelton D.A., Gavine A., Gray N.M., Toma M., Morris J. (2017). Promotion of physical activity interventions for community dwelling older adults: A systematic review of reviews. PLoS ONE.

[48] Overdorf V., Coker C., Kollia B. (2016). Perceived Competence and Physical Activity in Older Adults. Activ Adapt. Aging.

[49] Bandura A. (1986). Social Foundations of Thoughts and Actions.

